# Functional conservation between rodents and chicken of regulatory sequences driving skeletal muscle gene expression in transgenic chickens

**DOI:** 10.1186/1471-213X-10-26

**Published:** 2010-02-25

**Authors:** Michael J McGrew, Adrian Sherman, Simon G Lillico, Lorna Taylor, Helen Sang

**Affiliations:** 1 The Roslin Institute and Royal Dick School of Veterinary Studies, University of Edinburgh, Roslin, Midlothian, UK

## Abstract

**Background:**

Regulatory elements that control expression of specific genes during development have been shown in many cases to contain functionally-conserved modules that can be transferred between species and direct gene expression in a comparable developmental pattern. An example of such a module has been identified at the rat myosin light chain (*MLC*) 1/3 locus, which has been well characterised in transgenic mouse studies. This locus contains two promoters encoding two alternatively spliced isoforms of alkali myosin light chain. These promoters are differentially regulated during development through the activity of two enhancer elements. The *MLC3 *promoter alone has been shown to confer expression of a reporter gene in skeletal and cardiac muscle in transgenic mice and the addition of the downstream *MLC *enhancer increased expression levels in skeletal muscle. We asked whether this regulatory module, sufficient for striated muscle gene expression in the mouse, would drive expression in similar domains in the chicken.

**Results:**

We have observed that a conserved downstream *MLC *enhancer is present in the chicken *MLC *locus. We found that the rat *MLC1/3 *regulatory elements were transcriptionally active in chick skeletal muscle primary cultures. We observed that a single copy lentiviral insert containing this regulatory cassette was able to drive expression of a *lacZ *reporter gene in the fast-fibres of skeletal muscle in chicken in three independent transgenic chicken lines in a pattern similar to the endogenous *MLC *locus. Reporter gene expression in cardiac muscle tissues was not observed for any of these lines.

**Conclusions:**

From these results we conclude that skeletal expression from this regulatory module is conserved in a genomic context between rodents and chickens. This transgenic module will be useful in future investigations of muscle development in avian species.

## Background

The development of an organism entails the precise expression of lineage and tissue-specific gene products in a temporally-regulated manner during embryogenesis. The information for a cell to respond to external signals by differentiating down a particular developmental pathway is 'hardwired' into the regulatory regions surrounding these developmentally regulated genes [reviewed in [1]]. These conserved regulatory regions or modules drive spatial gene expression patterns in the forming tissues of the developing organism. Changes in the cis-regulatory elements of regulatory modules are hypothesized to be the predominant mechanism behind evolutionary changes in pattern formation [2].

Many expression modules have been shown to be functionally conserved in vertebrate species. For example, regulatory regions from several *hox *genes from fish and chicken are capable of driving some aspects of the spatial expression patterns of the paralogous murine gene in transgenic mice [3-6]. Examples of conserved regulatory modules have been shown for the processes of neurogenesis [7-9], limb morphogenesis [10] and haematopoiesis [11,12], amongst many other examples.

We, and others, have previously shown that lentiviral vectors can be used to generate transgenic chickens and that cis-regulatory regions incorporated into these vectors will drive ubiquitous or tissue-specific expression in this species [13-16]. In this report we investigate the possibility of utilising rodent regulatory elements to drive transgene expression in skeletal muscle of chickens. To achieve this we investigated the transcriptional activity of the rat *MLC *regulatory domains in transgenic chickens. This locus encodes two alkali myosin light chains expressed from two promoters that are differentially regulated during development. The MLC1 isoform is expressed at embryonic stages of development and in the fast fibres of skeletal muscles of the adult. The MLC3 isoform is expressed at fetal stages and in the atria of the mouse heart [17,18]. The construct we used consists of the rat *MLC3 *promoter, which is transcriptionally active in all striated muscle in mouse transgenic models [18,19], and a downstream rat *MLC *enhancer which augments skeletal muscle expression and confers expression at embryonic stages of development [20,21]. We show that a putative *MLC *enhancer is present in the chicken *MLC *locus. Using the rat *MLC *regulatory elements, we show that these elements support transgene expression in skeletal muscle of chickens. Cardiac transgene expression was not detected. These results indicate a functional conservation of the *MLC *regulatory elements exist between rodents and chickens in the skeletal muscle lineage. This demonstration is significant not only for the use of the chicken as a model organism for studies in developmental biology but also because poultry are an economically important food source.

## Results and Discussion

**Figure 1:**
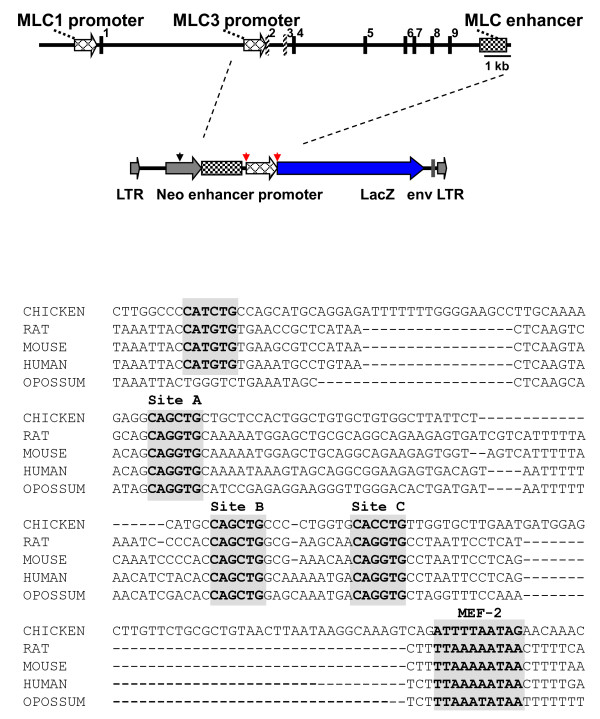
**The rat *MLC1/3 *locus and lentiviral construct**. Top: The rat *MLC *locus consists of two separate promoter elements which generate two alternatively spliced transcripts. A downstream enhancer augments expression from both promoters in skeletal muscle. Stippled exons are specific for *MLC3*.  The internal *MLC3 *promoter and the downstream enhancer were cloned upstream of a *lac*Z reporter construct in an EIAV replication defective lentiviral vector to generate pONY-MLZ. Restriction sites for *Hind*III (red arrows) and *Sph*1 (black arrows) are indicated.  Bottom: Sequence comparison of homologous downstream regions of the MLC1/3 locus of eutherans and chickens. Grey boxes highlight core enhancer elements identified in human, mouse, and rat. The internal basepairs of the E box of Site A are changed to the sequence of site B in the chicken. Site C is in reverse orientation in the chicken to that in mammals.

The mammalian *MLC *locus consists of two widely separated promoters driving expression of two protein isoforms of the alkali MLC and a downstream enhancer [20,22-24]. The exon structure of the chicken, rat, mouse, and human myosin light chain 1/3 locus is highly conserved [22-26]. (Fig. 1top). The rat and mouse *MLC *enhancer and the *MLC1 *promoters were previously shown to drive robust expression in fast skeletal muscle of transgenic mice [20,21]. Cardiac expression was shown to be dependant on the *MLC3 *promoter [18,19].

Examination of the chicken locus at an analogous position for the *MLC *enhancer enabled us to identify a region of homology shared with human, rat, mouse, and opossum (Fig. 1bottom). A closer examination of this region revealed a putative enhancer module containing regulatory elements that closely resemble the core myosin light chain enhancer as defined in mouse, rat and human [19,27,28]. This core enhancer contains three canonical E boxes (CANNTG) that bind to the myogenic factors, myf-5, MRF4, myoD, and myogenin and an A/T rich region containing a sequence matching the consensus binding site of MEF2 [27]. All four regulatory motifs are present in this DNA sequence as indicated in Fig. 1. These data suggest that the structure of the *MLC1/3 *locus is highly conserved between mammals and chicken.

### Generation of a muscle-specific reporter cassette

**Figure 2:**
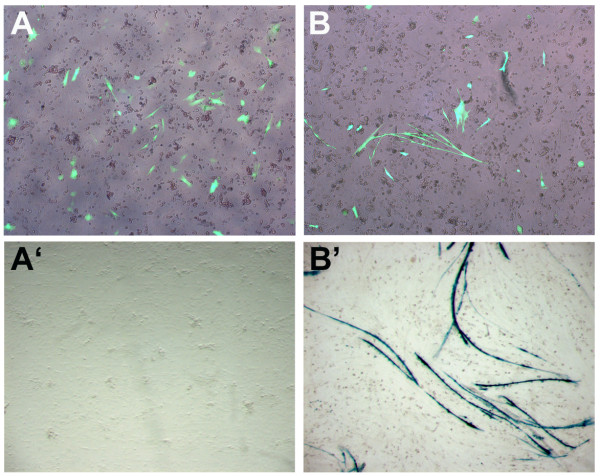
***MLC3 *regulatory elements are transcriptionally active in chicken skeletal muscle but not cardiac muscle primary cultures**. Cardiac (A, A') and skeletal (B, B') muscle chick primary cultures were co-transfected with the MLC3 lentiviral construct and a CAG-GFP reporter construct. Three independent experiments were carried out and a representative field is shown. A, B: GFP fluorescence A'B': X-gal staining of primary cultures. Large myotubes are stained in B'.

To define a regulatory module that is sufficient for gene expression in the striated muscle of transgenic chickens, we used the regulatory regions from the rat *MLC1/3 *locus to drive expression of a *lacZ *reporter gene. These elements were both introduced upstream of the *lacZ *reporter gene in a minimal EIAV replication-defective lentiviral vector to create the construct pONY-MLZ (Fig. 1C). To test the transcriptional activity of the lentiviral construct in chicken cells, transient transfections of the construct were carried out into primary cultures of embryonic chicken cardiac and skeletal muscle cells and the transfected cells were stained for β-galactosidase activity (Fig. 2). An upstream CMVenhancer/promoter regulatory region used for packaging the lentiviral construct was first removed from this plasmid to avoid possible transactivation of the downstream *MLC3 *promoter (see Methods). A GFP reporter construct was co-transfected to visualise transfection efficiency into these cells (Fig. 2A, 2B). Transfections into skeletal muscle primary cultures demonstrated that long myotubular cells stained positive for β-galactosidase whilst surrounding fibroblasts did not (Fig. 2B'). In transfections into cardiac muscle primary cultures, no expression of β-galactosidase was detected (Fig. 2A'). These results indicate that the *MLC *regulatory module is transcriptionally active in skeletal muscle cells but is not active in cardiac muscle. We went on to test this module *in vivo *using transgenesis.

### Generation of transgenic chickens carrying the MLC-*lacZ *transgene

**Figure 3:**
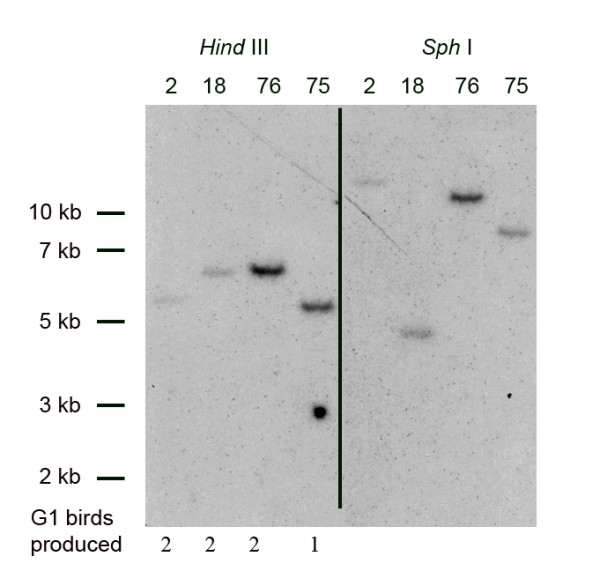
**Southern blot analysis of transgenic G_1 _chickens**. Genomic DNA from birds containing the MLZ proviral sequences were analysed for integration events by digestion with *Hind*III (left) or *Sph*1 (right) to generate junction fragments. The number of G_1 _birds generating identical junction fragments is indicated.

To generate transgenic chickens containing a MLC transgene, a concentrated preparation of pONY-MLZ replication-defective lentivirus was produced. The virus was injected into the sub-germinal cavity of fertilised eggs at the new-laid egg stage. Injected eggs were incubated until hatching using the surrogate shell culture system as described [13]. A total of 12 chickens were hatched and all were identified as transgenic for the EIAV vector by semi-quantitative PCR of DNA from blood samples. One cockerel, estimated to have an incidence of 10% transgene integration in the germline by PCR analysis of semen DNA, was bred with stock hens and G_1 _transgenic offspring identified. Seven G_1 _offspring were produced and genomic DNA from these birds analyzed by restriction enzyme digestion and Southern blot analysis to determine the number of vector insertions in each bird and the number of different insertion events represented in the G_1 _generation. Genomic DNA digested with *Hind*III and *Sph*1 generated junction fragments that revealed that all seven birds contained single vector insertions and four independent insertions were present in the G_1 _birds (Fig. 3). Four G_1 _birds, each carrying a different transgene integrant, were bred to generate G_2 _birds which were analysed in the subsequent experiments.

### Transgene expression is limited to avian skeletal muscle

**Figure 4:**
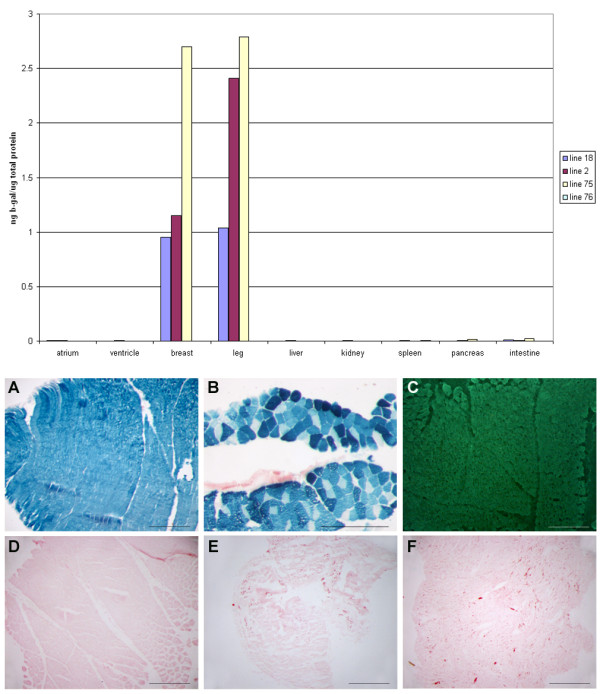
**Transgene expression is limited to skeletal muscle**. Top: Protein samples from various tissues were analysed by ELISA for the presence of β-galactosidase. Each data point is the average of two independent assays on tissues from one transgenic bird for each line. Birds were a minimum of four months of age when assayed. Line 76 is not visible on the graph due to the low expression levels of this line. Bottom: Sections from various tissues of the same birds were stained for β-galactosidase activity. A) Transgenic breast muscle. B) Transgenic leg muscle. C) Immunofluorescence of the endogenous MLC1 and 3 protein D) Control breast muscle. E) Transgenic atrium. F) Transgenic ventricle. Staining in skin appears to be in striated muscles associated with feather tracts. Scale bars, 0.5 mm.

To determine the transcriptional profile of the *MLC *expression module in transgenic chickens, selected tissues from chickens carrying each of the four independent vector insertions were assayed for β-galactosidase protein by ELISA (Fig. 4, top). β-galactosidase protein was detected in breast and leg muscle of three transgenic lines (lines 2, 18, 75). The skeletal muscle expression levels were relatively robust. For example in line 75, 0.25% of the total protein in skeletal muscle was β-galactosidase. Non-muscle tissues did not contain β-galactosidase. No β-galactosidase was detected in tissue samples from the fourth transgenic line (line 76). It is possible that the integrated vector for this line has acquired a mutation that inactivated the transgene or that it has integrated into a transcriptionally silent site.

Unexpectedly, no β-galactosidase was detected in heart atria or ventricles of birds from any of the three expressing lines. In transgenic mice, the *MLC3 *promoter alone drove β-galactosidase expression in cardiac muscle of transgenic mice (3 of 3 mouse lines) [18]. Addition of the *MLC *enhancer to the transgene construct inhibited expression in cardiac tissues of two out of three transgenic mouse lines. A similar effect was also reported by Zammit et al., [21]. The lack of expression in cardiac tissue from the transgenic chickens mimicked the result obtained by transfection experiments into chicken cardiomyocytes and was also similar to the expression patterns observed in transgenic mice for constructs containing the *MLC *enhancer.

Tissues from the transgenic adult birds were stained for β-galactosidase activity to observe the spatial expression pattern in skeletal muscle. Non-muscle tissues and cardiac muscle did not stain for β-galactosidase (Fig. 4, bottom). Pectoral muscle of the breast was entirely stained for β-galactosidase. Immunofluorescence of chicken pectoral muscle with an antibody to chicken myosin light chain 1 and 3 revealed extensive expression throughout this tissue (Fig. 4C). In contrast to the pectoral musculature, staining of leg muscle displayed a cobbled expression pattern. Chicken pectoral muscle contains predominantly fast fibre muscle (white muscle) whereas leg musculature contains both fast and slow muscle fibres (red muscle) [29,30]. This result suggests that the transgene is expressed extensively in the fast fibre tissue of the breast flight muscle and is restricted in expression in leg muscle which contains slow as well as fast muscle fibres.

**Figure 5:**
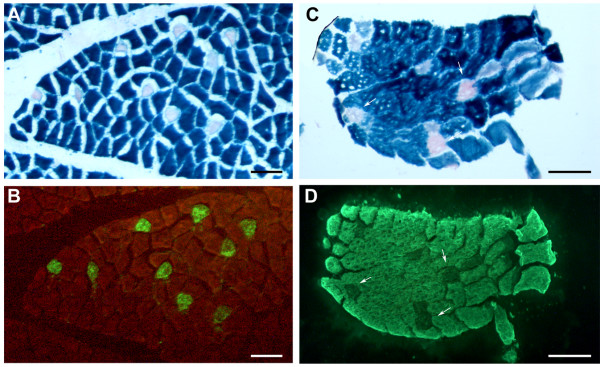
**Transgene expression is restricted to fast skeletal muscle fibres and is expressed in a similar fibre type as the endogenous locus**. A) A section from an interior leg muscle of a transgenic chicken was stained for β-galactosidase activity. The animal was at least four months old when assayed. B) A serial section to the one above was immuno-stained for fast (red) and slow (green) muscle isoforms. Fibres that are negative for β-galactosidase are labelled with an antibody to slow muscle.  C) A section from an interior leg muscle of a second line of transgenic chicken was stained for β-galactosidase activity. D) A serial section to the one above was immuno-stained for MLC1 and 3 muscle isoforms (green). Fibres that are negative for β-galactosidase do not express MLC1 and 3. White arrows indicate corresponding myofibres. Scale bars, 0.1 mm.

Tissue from the leg muscle was stained for β-galactosidase and an adjacent section immunostained with fast and slow fibre-specific antibodies [31], to demonstrate that the transgene expression is limited to fast fibres. The results shown in Fig. 5A and 5B demonstrate that the fibres that stained for β-galactosidase activity were also labelled with the fast fibre marker, F59. Conversely, fibres negative for β-galactosidase activity were immuno-positive for the slow fibre marker, S58. This finding is in accordance with results obtained in mouse models, which demonstrated that the *MLC1/3 *regulatory module conferred expression to fast muscle fibres [18,19,32].

To demonstrate that the transgene expression follows that of the endogenous *MLC1/3 *locus, a tissue section was stained for β-galactosidase and an adjacent section immunostained with an antibody to MLC1 and 3 isoforms (F310) (Fig. 5C, 5D) [33]. Muscle fibres that are negative for β-galactosidase activity are immuno-negative for MLC1 and 3 indicating that the rat regulatory elements are expressed in a similar fibre type as the endogenous gene.

### The *MLC *module drives early embryonic expression

**Figure 6:**
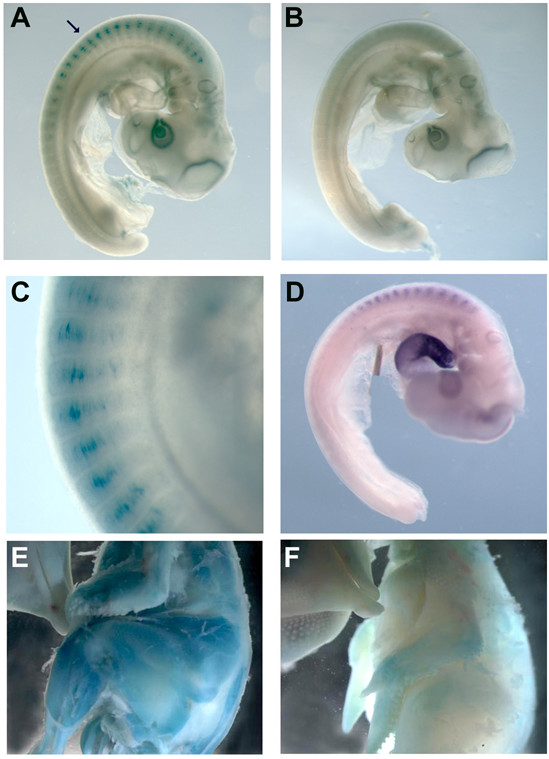
**Transgene expression in the forming chicken embryo**. Transgenic and control embryos were stained for of β-galactosidase activity. A) Transgenic and (B) control embryos at day 3 of development. C) Higher magnification of the embryo in (A) to show staining in the forming myotome. D) Transcripts from the endogenous locus at day 3 are revealed by in situ hybridisation for both *MLC1 *and *3*. E) Transgenic and (F) control embryos at day 10 of development. Arrow, myotome.

We next examined the temporal expression pattern of the MLC construct at selected embryonic stages and compared these expression patterns with those of the endogenous locus, to determine if the transgenic construct is accurately developmentally regulated. Staining of chicken embryos for β-galactosidase activity revealed that the MLC transgene was expressed in the forming myotome of day 3 (HH stage 19) chicken embryos (Fig. 6A-6C). *In situ *hybridisation with a riboprobe specific for *MLC1 *and *3 *mRNA also revealed that the endogenous chicken locus is transcribed in the chicken myotome at this stage (Fig. 6D). Abundant staining of the cardiac tube was also apparent. An analysis of later stages revealed extensive staining of β-galactosidase activity through the forming muscle masses of day 10 (mid-incubation) transgenic chicken embryos (Fig. 6E, 6F). These analyses show that the MLC regulatory module is sufficient to drive embryonic expression in skeletal muscles of chicken but not in cardiac tissue. Transgenic constructs containing these regulatory elements had previously been shown to be expressed in the myotome of developing transgenic mouse embryos [18,19,21]. Thus, our results indicate a conservation of temporal expression patterns between rodents and chicken.

## Conclusion

These experiments are a further demonstration that the chicken can be used as an alternative model for transgenic analysis of gene regulation and of the use of lentivirus in avian transgenesis. These results indicate that the MLC regulatory module is functionally conserved for fast muscle fibre expression in mammals and birds and is developmentally regulated. Regulatory regions in the MLC enhancer are also conserved between mammals and chicken (Fig. 1). In contrast, sequences in the *MLC3 *promoter are divergent when compared to the *MLC1 *promoter [18,25]. The GATA regulatory element of the rat *MLC3 *promoter, essential for cardiac expression in the rat, is not conserved in the chicken MLC3 promoter, yet there is expression from the endogenous *MLC1/3 *locus in the embryonic chicken heart (Fig. 6D), suggesting that regulatory modules for avian cardiac expression are divergent from those in mammals.

The construct described here will be useful as an expression module to drive skeletal muscle expression in avian species. This will have applications for the expression of proteins or RNAs in avian muscle tissue for studies of muscle differentiation and function. For example, the expression of microRNAs targeting genes involved in skeletal muscle myopathy in broiler chickens could be specifically targeted to skeletal muscle using this regulatory module [34]. Additionally, similarly constructed transgenes will permit the analysis of candidate genes, identified by QTL mapping and expression analyses, implicated in control of muscle mass in chicken lines selected for increased muscle mass [35,36]. Inherent difficulties still remain in the generation of transgenic chickens including the long period to sexual maturity (16 weeks) and the difficulties in generating high titre lentivirus for large and complex transgenes.

## Methods

### Lentivirus construction and packaging

The 628 bp MLC3 promoter was isolated from p628Luc920 [18] using *Hind*III and subcloned into the *HindIII *site of the minimal EIAV lentiviral vector pONY8.45NCmcs [15]. A *lacZ *reporter gene was isolated from pONY 8.45NCZ using *Sph*1 and *Xho*1 and subcloned into the *Xho*1 and *Nru*1 sites of the above plasmid to generate pONY8.45NCMLC3-Z. A residual CMV promoter was deleted from this vector by digestion with *Eco*R1 and *Sac*II and re-ligation of the plasmid. Finally, a 920 bp MLC enhancer fragment was isolated from p628Luc920 [18] using *Bam*H1 and subcloned into the *Not*1 site upstream of the introduced promoter fragment to generate pONY8.45N-E-MLC3-Z (pONY-MLZ). The original direction of the enhancer was reversed in this construct. Packaged virus stocks were generated by FuGENE6 (Roche, Lewes, U.K.) transfection of HEK 293T cells plated on 10 cm dishes with 2 μg of vector plasmid, 2 μg of gag/pol plasmid (pONY3.1) and 1 μg of VSV-G plasmid (pRV67) [37]. 36-48 hours after transfection supernatants were filtered (0.22 μm) and stored at -80°C. Concentrated viral preparations were made by initial low speed centrifugation at 6,000 × g for 16 hours at 4°C followed by ultracentrifugation at 50,500 × g for 90 minutes at 4°C. The virus was resuspended in formulation buffer (20 mM Tris, 100 mM NaCl, 10 mg/ml sucrose,10 mg/ml mannitol, pH 7.4) for 2-4 hours, aliquoted and stored at -80°C. Viral titre was estimated at 4.5 × 10^7 ^T.U./μl.

### Production and analysis of transgenic birds

Founder transgenic birds were generated by injection of viral particles into fertilised eggs at the new-laid egg stage, followed by culture of the embryos to hatch as described [13,38]. The hatched chicks were raised to; sexual maturity and DNA extracted from semen from adult males was screened by PCR (13) to; identify cockerels that carried vector sequences in the germ line. One cockerel identified by this method was crossed to; stock hens and their offspring screened by PCR to; identify G_1 _hemizygous transgenic birds. All experiments, animal breeding, and care procedures were carried out under license from the U.K. Home Office.

The vector insertions in individual G_1 _birds were analyzed by Southern transfer. Genomic DNA (10 μg), extracted from whole blood, was digested with *Hind*III or *Sph*1 restriction endonucleases, resolved on a 0.7% agarose gel, and transferred to Hybond-N membrane (Amersham Biosciences, U.K.). These blots were analyzed by using a probe of the *lac*Z coding sequence, labelled with [^32^P]dCTP using a RediPrime II kit (Amersham Biosciences). Hybridization was in 500 mM sodium phosphate (pH 7.2)/7% SDS at 65°C and signal was detected by autoradiography.

### Transfection of chicken primary cultures

The lentiviral plasmids described above were digested with *Bgl *II and *Xba *I and re-ligated to remove a 1.8 kb CMV enhancer/promoter fragment used for transcribing the lentiviral constructs during packaging and which could potentially transactivate the downstream *MLC3 *promoter (pONY-MLZ-del CMV constructs).

Skeletal or cardiac tissue was isolated from day 9 chicken embryos, macerated and dissociated in trypsin for 3 × 5 minutes with vigorous pipetting. The solution was spun at 1000 × g, 5 minutes, and re-suspended in plating media (10%FBS, 1% chick serum in DMEM, 1× NEAA). On day two of culture, cells were transfected using Fugene HD (Roche) with 5 ug of pONY-MLZ (del CMV) constructs and 1 ug of CAG-GFP in 35 mm tissue culture plates (Primaria, BD Biosciences). Culture media for skeletal muscle cultures was changed to differentiation media (2% chick serum in DMEM, 1× NEAA) after 24 hours. All plates were incubated for two additional days, photographed for GFP fluorescence, and stained for β-galactosidase activity as described below.

### Analysis of β-galactosidase activity and processing of tissues

Selected tissues were snap-frozen and total protein extracted by homogenization in PBS containing protease inhibitors (complete mini, Roche). Protein concentration was determined by Bradford assay. ELISA was performed using β-gal Elisa kit (Roche). Elisa assays were carried out on two G_1 _birds for each integration event. The data shown in Fig. 4 are for one transgenic animal for each integration event.

Adult tissues were isolated, fixed for 30 min in 4% paraformaldehyde, 0.25% gluteraldehyde in phosphate buffered saline (PBS) and tissues cryo-embedded and sectioned at 14 μm. β-galactosidase activity was detected by incubating at 37°C in 5 mM potassium ferricyanide, 5 mM potassium ferrocyanide, 2 mM MgCl_2_, 0.5 mg/ml X-gal for 90 minutes, counterstained with eosin and mounted. Fast (F59) and slow (S58) fibre-type antibodies and an antibody against MLC1 and 3 isoforms (F310), developed by F.E. Stockdale, were obtained from the Developmental Studies Hybridoma Bank, University of Iowa, and used at 1:10 dilutions. The secondary antibodies used were TRITC conjugated goat anti-mouse IgG and FITC conjugated goat anti-mouse IgA (Sigma), and Alexa Fluoro 488 conjugated goat anti-mouse IgG respectively. Slides were counterstained with Hoechst, and mounted with Vectashield mounting media (Vector). Whole mount in situ hybridizations were carried out as described [39]. The riboprobe to MLC1 and 3 transcripts was described in [18].

## Authors' contributions

MJM and HS conceived and designed the experiments. SGL produced and titred the virus. AS produced the transgenic birds. LT cloned several of the plasmid constructs. MJM performed most of the analysis. MJM wrote the manuscript with subsequent contributions from all authors. All authors read and approved the final manuscript.
